# Supplementary material for: VEGFR2 but not VEGFR3 governs integrity and remodeling of thyroid angiofollicular unit in normal state and during goitrogenesis
Source: EMBO Mol Med. 2017 Apr 24;9(6):750–69. doi: 10.15252/emmm.201607341 (PMC5452036; doi:10.15252/emmm.201607341)
Supplement: Supplementary file 1 — Appendix [file EMMM-9-750-s001.pdf]

Appendix

VEGFR2 but not VEGFR3 governs integrity and remodeling of thyroid angiofollicular unit in normal state and during goitrogenesis

Jeon Yeob Jang, Sung Yong Choi, Intae Park, Do Young Park, Kibaek Choe, Pilhan Kim, Young Keum Kim, Byung-Joo Lee, Masanori Hirashima, Yoshiaki Kubota, Jeong-Won Park, Sheue-Yann Cheng, Andras Nagy, Young Joo Park, Kari Alitalo, Minho Shong & Gou Young Koh

Table of Contents:

Appendix Figures S1-S6

Appendix table S1

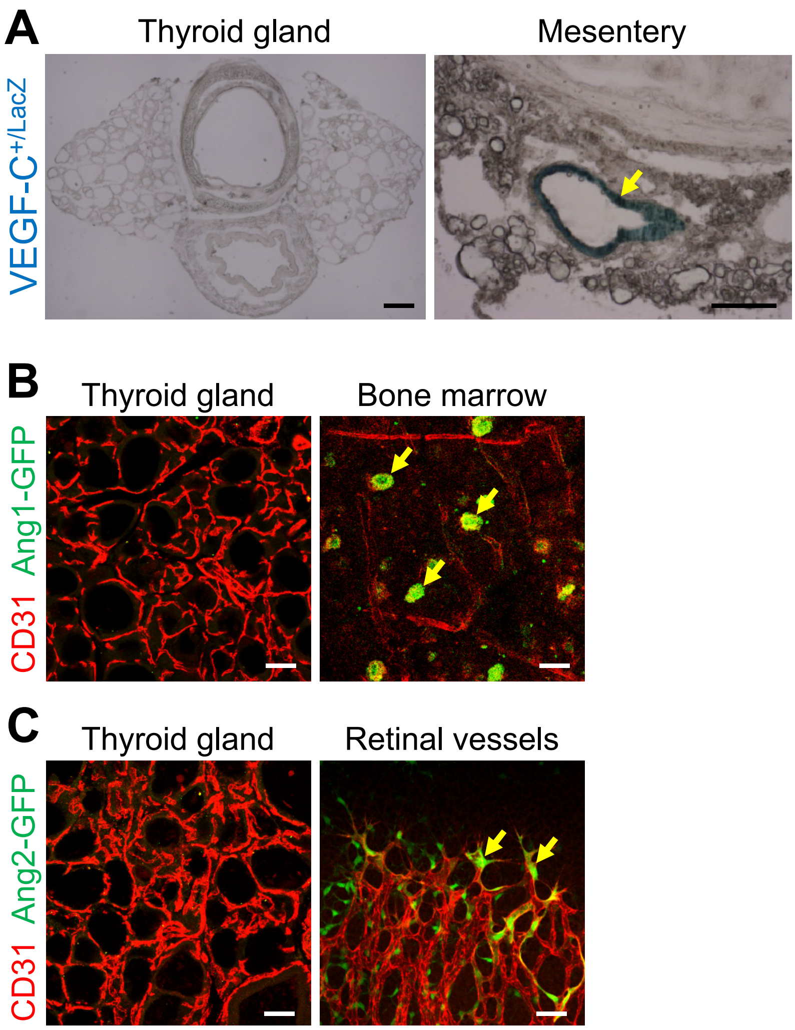

Appendix Figure S1. Expression patterns VEGF-C, Ang1 and Ang2 in thyroid gland and other organs.

A Images showing VEGF-C expression in the thyroid gland and mesentery of adult *VEGF-C*$^{+/LacZ}$ mouse. Yellow arrow indicates the expression of VEGF-C in mesenteric lymphatic vessel as a positive control. Scale bars, 200 µm.

B Images showing Ang1 expression in thyroid glands and megakaryocytes (yellow arrows) in bone marrow of Ang1-GFP reporter mouse at adulthood. Scale bars, 50 µm.

C Images showing Ang2 expression in thyroid glands at adulthood and tip ECs (yellow arrows) of growing retinal vessels at P5 of Ang2-GFP reporter mice. Scale bars, 50 µm. Similar results were obtained from 3-4 different mice for each experiment.

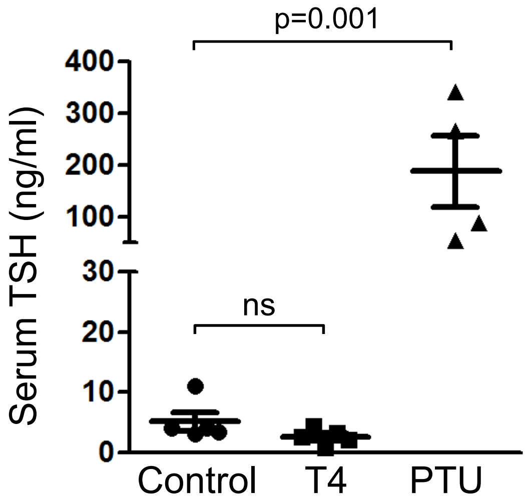

Appendix Figure S2. Serum concentrations of TSH in control or T4- or PTU-treated mice. Error bars represent mean±s.d. Kruskal-Wallis test followed by Turkey’s multiple comparison test was used. ns, not significant.

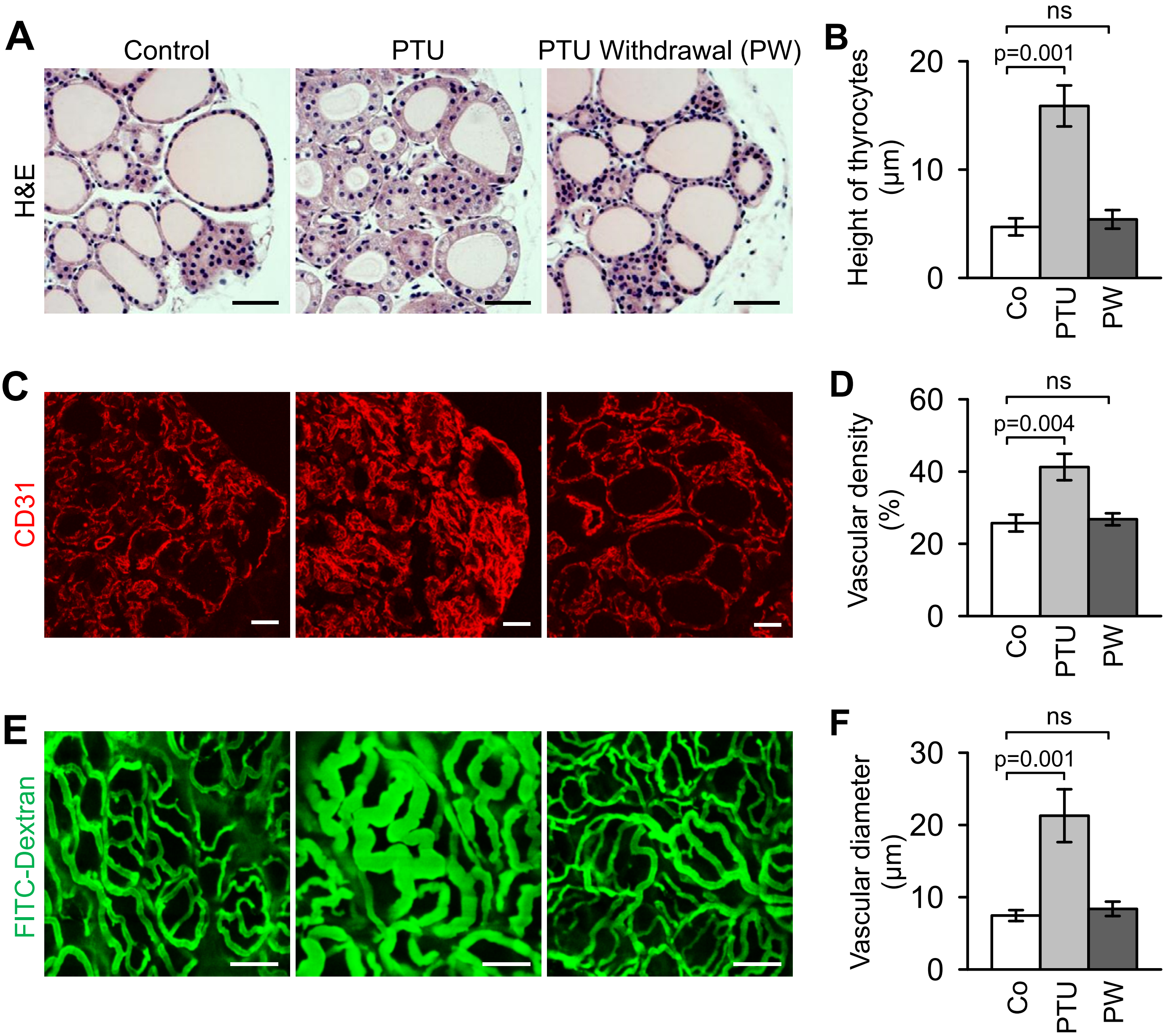

Appendix Figure S3. PTU-induced thyroid angiofollicular remodeling is a reversible process.

The adult mice were treated with control (Co), PTU for 3 weeks. The thyroid glands were sampled and analyzed 3 weeks after the withdrawal of PTU administration (PW).

A-F Images and comparisons of height of thyrocytes, $CD31^+$ BV density, and intravital FITC-dextran perfused BV diameter in control (Co), PTU-treated (PTU) or PTU-withdrawal (PW) mice. All scale bars, 50 µm. Each group, n = 4. Error bars represent mean±s.d. Kruskal-Wallis test followed by Turkey's multiple comparison test was used. ns, not significant.

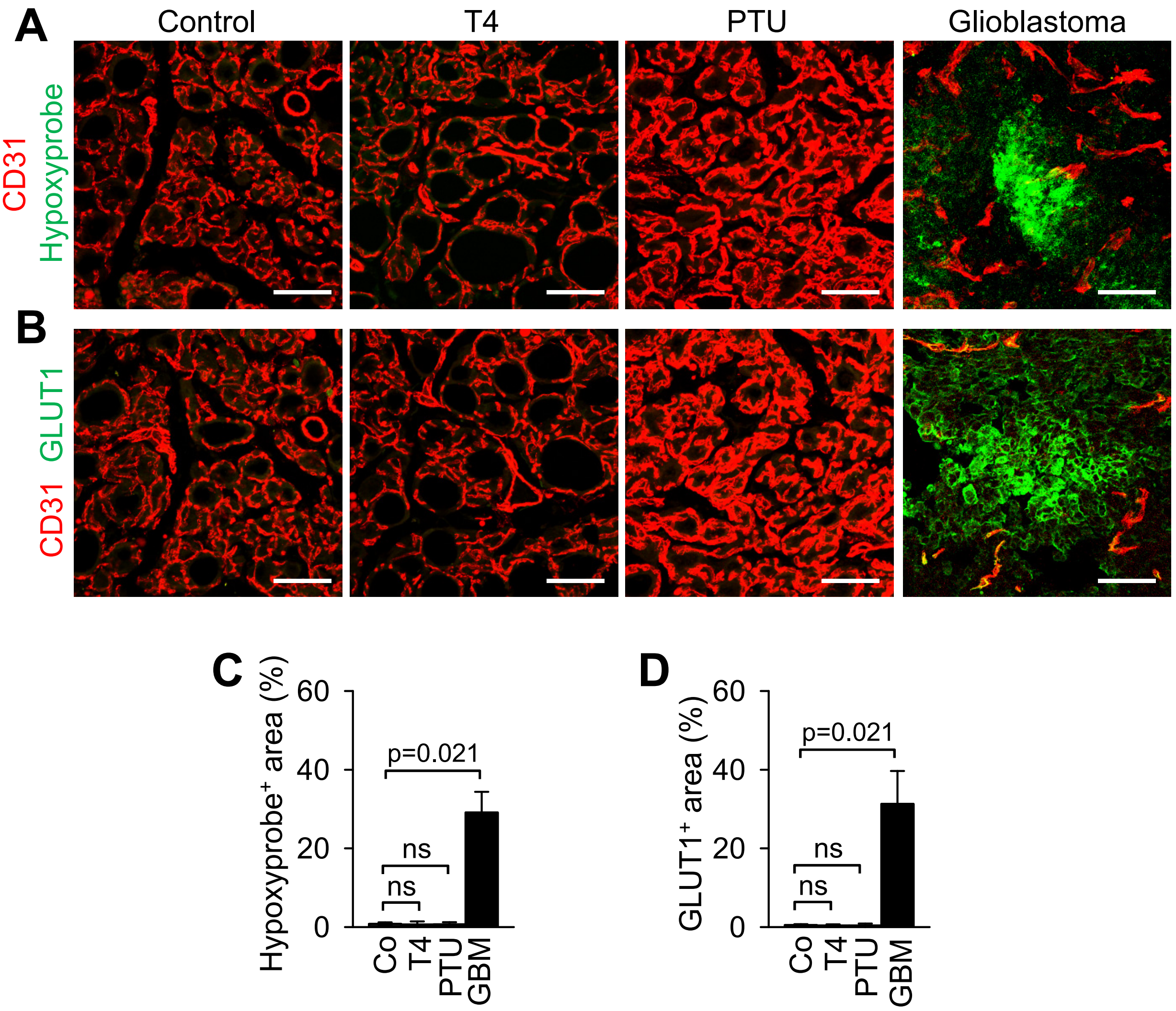

Appendix Figure S4. Tissue hypoxia is not detected in thyroid glands of control, T4-treated, PTU-treated mice.

A-D Images and comparisons of tissue hypoxia status (Hypoxyprobe, GLUT-1) in thyroid glands of control (Co), T4-treated (T4), PTU-treated (PTU) mice and implanted gloiblastoma tumor (GBM, positive control). Scale bars, 50 µm. Error bars represent mean±s.d. Each group, n = 4. Kruskal-Wallis test followed by Turkey's multiple comparison test was used. ns, not significant.

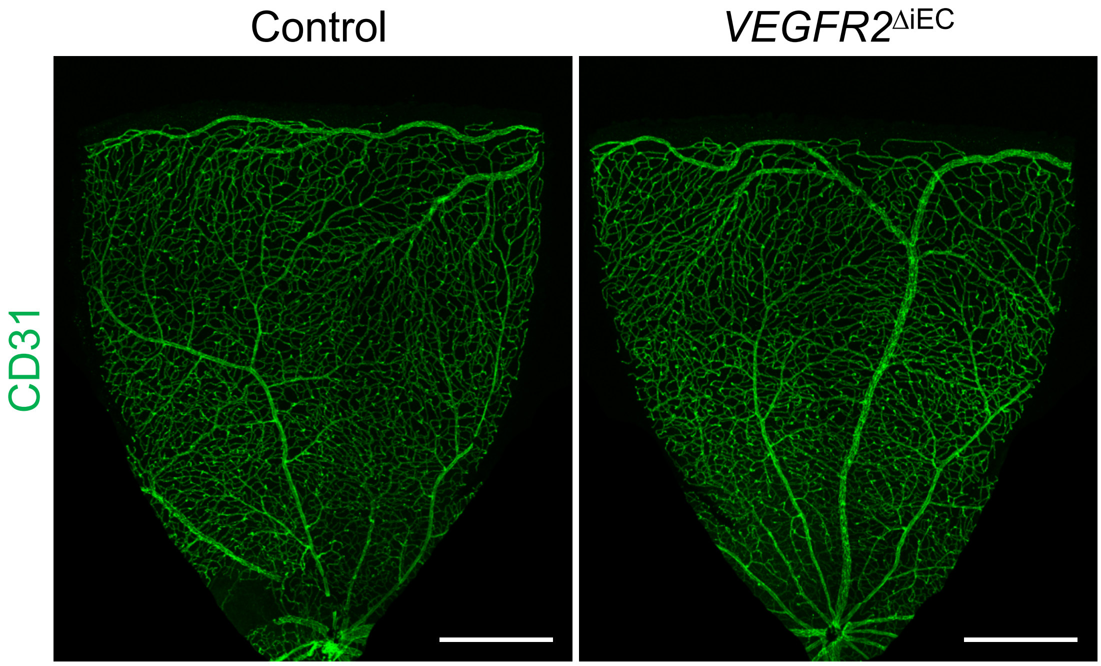

Appendix Figure S5. Images showing retinal blood vessels in control and *VEGFR2*$^{\Delta iEC}$ mice after tamoxifen administration at adulthood. Scale bars, 500 µm.

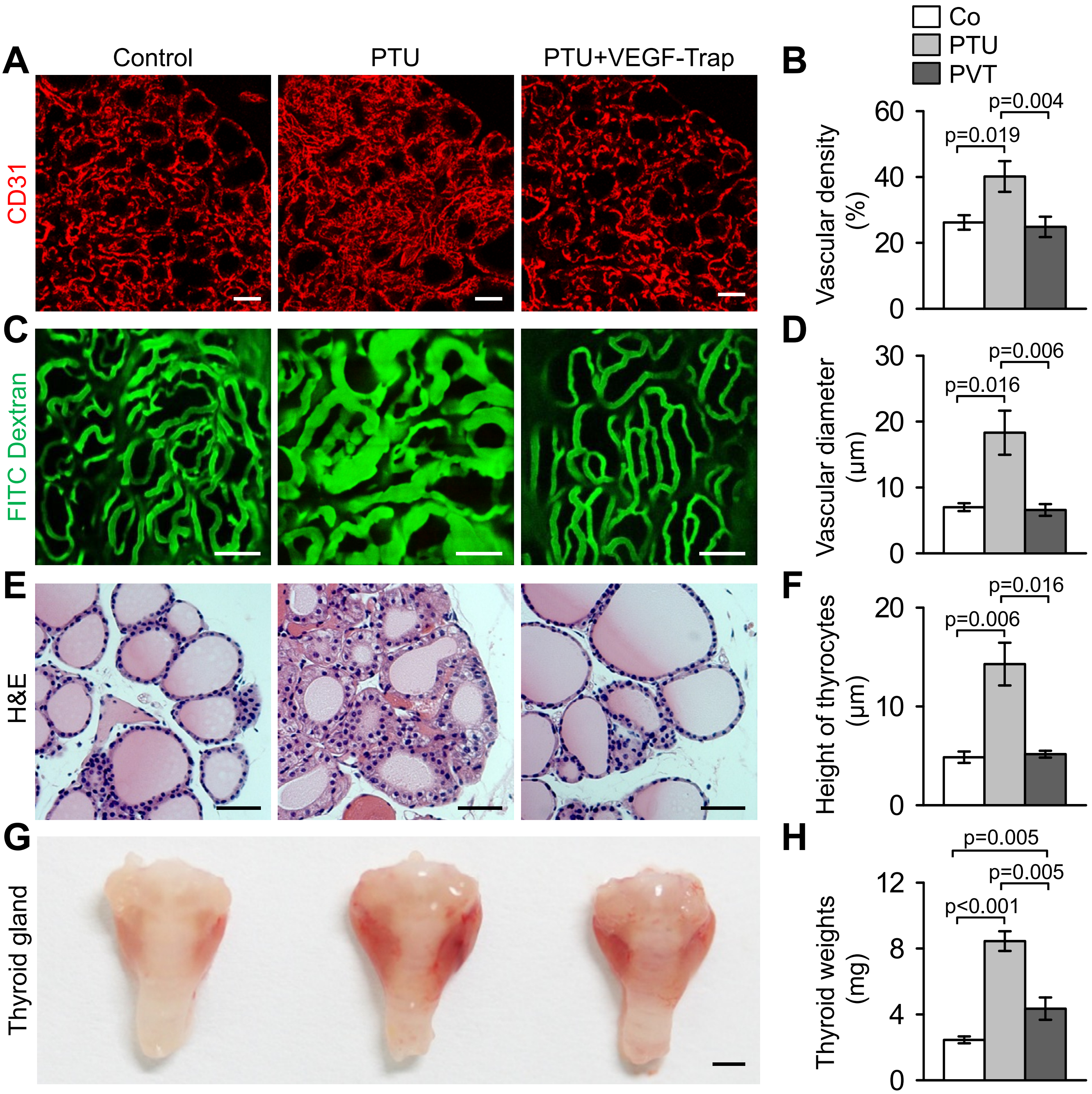

Appendix Figure S6. Blockade of VEGF-A ameliorates angiofollicular remodeling during PTU-induced goitrogenesis.

A-F Images and comparisons of CD31^{+} BV density, intravital FITC-dextran perfused BV diameter, and height of thyrocytes in thyroid glands of control (Co), PTU-treated (PTU), or PTU+VEGF-Trap-treated (PVT) mice. Scale bars, 50 µm. Error bars represent mean±s.d. Each group, n = 4. Kruskal-Wallis test followed by Turkey's multiple comparison test was used.

G,H Gross image of thyroid glands and comparisons of thyroid weights in control (Co), PTU-treated (PTU), or PTU+VEGF-Trap-treated (PVT) mice. Scale bars, 1mm. Error bars represent mean±s.d. Each group, n = 4. Kruskal-Wallis test followed by Turkey's multiple comparison test was used.

Appendix Table S1

Appendix table S1. Primer sets for semi-quantitative RT-PCR or quantitative real-time PCR analysis.

Name	Sequence (5'-3')	
β-actin (housekeeping gene)	Forward	5'-GCTCTTTTCCAGCCTTCCTT-3'
	Reverse	5'-CTTCTGCATCCTGTCAGCAA-3'
VEGF-A (for RT-PCR)	Forward	5'-CAGGCTGCTCTAACGATGAA-3'
	Reverse	5'-CAGGAATCCCAGAAACAACC-3'
VEGF-A_{164}	Forward	5'-CTCCACCATGCCAAGTGGTC-3'
	Reverse	5'-TCGTTACAGCAGCCTGCACA-3'
VEGFR2	Forward	5'-ACCAGAAGTAAAAGTGATCCCAGA-3'
	Reverse	5'-TCCACCAAAAGATGGAGATAATTT-3'
VEGF-C	Forward	5'-GACATGTCCAACAAACTATGTGTGG-3'
	Reverse	5'-CTGTTACCATGGTCCCACAGAG-3'
VEGF-D	Forward	5'-GCCAGTATGGACTCACG-3'
	Reverse	5'-CAATTATCAGAAGATCC-3'
Ang1	Forward	5'-TAGAGCTACCAACAACAACAGCA-3'
	Reverse	5'-CCCTTTAGCAAAACACCTTCTTT-3'
Ang2	Forward	5'-CTCCAAGAGCTCGGTTGCTATCCG-3'
	Reverse	5'-GGCCTTGATCTCCTCTGTGGAGTTG-3'
PDGF-A	Forward	5'-CTGCTCCTCGGCTGCGGATACCTC-3'
	Reverse	5'-GAGTCGCTGGAGGTCCCGGATGCTG-3'
PDGF-B	Forward	5'-CACAGAGACTCCGTAGATGAAGATGGG-3'
	Reverse	5'-CACTCGGCGATTACAGCAGGCTCTG-3'
Pax8	Forward	5'-ATGCCTCACAACTCGATCAGATCCG-3'
	Reverse	5'-TGCCAAGGATCTTGCTTACACAGCC-3'
TPO	Forward	5'-GCCACCAAGATGTCCTGACACCTG-3'
	Reverse	5'-GGCAGTGGGAAGCCGTGGTATAAG-3'
NIS	Forward	5'-GCCAACACTTCCAGAGGGATCCC-3'
	Reverse	5'-TTGGGGCCACAGATGCTGTCTGC-3'
