# Supplementary material for: VEGFR2 but not VEGFR3 governs integrity and remodeling of thyroid angiofollicular unit in normal state and during goitrogenesis
Source: EMBO Mol Med. 2017 Apr 24;9(6):750–69. doi: 10.15252/emmm.201607341 (PMC5452036; doi:10.15252/emmm.201607341)
Supplement: Supplementary file 6 — Source Data for Figure 1 [file EMMM-9-750-s005.pptx]

## Slide 1
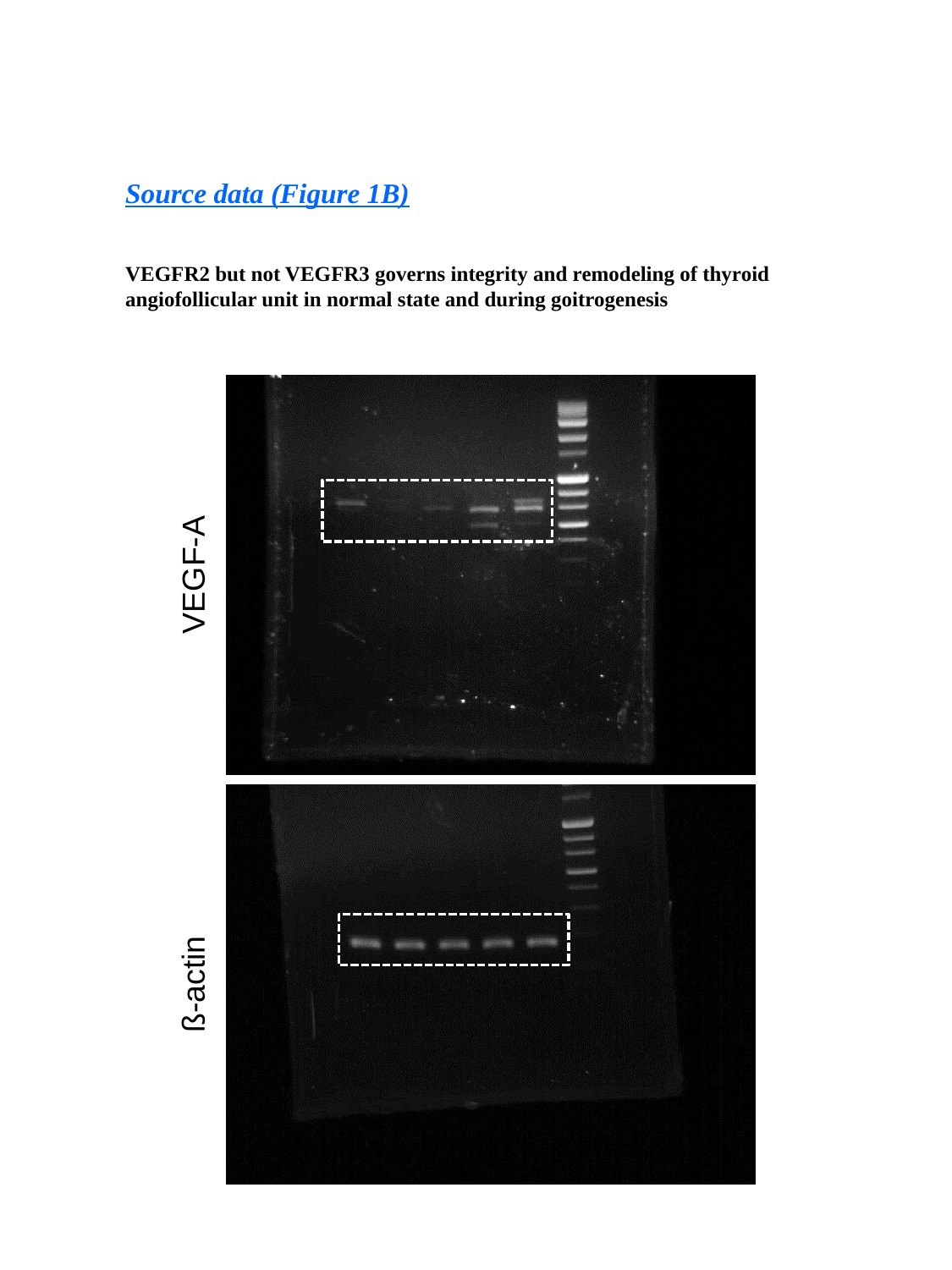

Source data (Figure 1B)
VEGFR2 but not VEGFR3 governs integrity and remodeling of thyroid angiofollicular unit in normal state and during goitrogenesis
VEGF-A
ß-actin
